# Cryptographic Keys Generating and Renewing System for IoT Network Nodes—A Concept

**DOI:** 10.3390/s20175012

**Published:** 2020-09-03

**Authors:** Janusz Furtak

**Affiliations:** Faculty of Cybernetics, Military University of Technology, 00-908 Warsaw, Poland; janusz.furtak@wat.edu.pl; Tel.: +48-261-838-701

**Keywords:** key distribution system, cryptographic keys renewing, security in IoT, Trusted Platform Module, MQTT secure data exchange

## Abstract

Designers and users of the Internet of Things (IoT) are devoting more and more attention to the issues of security and privacy as well as the integration of data coming from various areas. A critical element of cooperation is building mutual trust and secure data exchange. Because IoT devices usually have small memory resources, limited computing power, and limited energy resources, it is often impossible to effectively use a well-known solution based on the Certification Authority. This article describes the concept of the system for a cryptographic Key Generating and Renewing system (KGR). The concept of the solution is based on the use of the hardware Trusted Platform Module (TPM) v2.0 to support the procedures of creating trust structures, generating keys, protecting stored data, and securing data exchange between system nodes. The main tasks of the system are the secure distribution of a new symmetric key and renewal of an expired key for data exchange parties. The KGR system is especially designed for clusters of the IoT nodes but can also be used by other systems. A service based on the Message Queuing Telemetry Transport (MQTT) protocol will be used to exchange data between nodes of the KGR system.

## 1. Introduction

System designers in which security issues are an important element often use cryptographic techniques to secure stored data and to secure data exchange between system components. The cryptographic algorithms available on the market can be divided into two classes: asymmetric key algorithms and symmetric key algorithms. Each of these classes has different properties and applications. System designers are forced to solve the dilemma of which algorithms to use in a case. In most situations, mixed solutions are used.

Asymmetrical cryptography is used to build trust structures, to sign messages forwarded, and to establish a secure connection with the other party to exchange data. Using asymmetrical cryptography, mechanisms for distribution of symmetric keys are constructed, e.g., according to the Diffie–Hellmann scheme [1]. Asymmetric cryptography is rather not used to encrypt large data, because these algorithms are characterized by a relatively large overhead in relation to encrypted data, are time consuming and resource consuming, and to ensure adequate cipher strength these keys must be relatively long.

Symmetric cryptography is more computationally effective and provides adequate cipher strength by using keys much shorter than asymmetric cryptography. It is estimated that similar and today acceptable cipher power can be obtained for asymmetric cryptography, e.g., RSA for a key length of 3072 bits and for symmetrical cryptography for a key length of 128 bits [2]—the key length difference is very visible. However, there is a significant problem with symmetric cryptography. It is a distribution of the key. For this purpose, asymmetrical algorithms are usually used to establish a connection and determine the so-called session key, which is a symmetric key used by both sides of the connection. Further data exchange is already performed using the session key.

Each cryptographic key must be changed from time to time to make it difficult to guess the key by attackers. The criteria for determining the key validity are different [3]. Usually, one of two scenarios are used. The first scenario is based on the volume of data protected with this key, and the second one is based on the key usage time determined e.g., as the number of processed frames or as the elapsed time counted in seconds from the moment the key was generated. Regardless of how the key validity criterion is determined, different strategies for renewing cryptographic keys are used. The first group of methods uses asymmetric cryptography (very similar to the procedure for setting the session key). In the second group of methods, the “*old*” symmetric cryptographic key is used to support the procedure for obtaining the “*new*” key. In the third group of methods, when establishing the session key, an additional symmetric key is generated, which in the future will only be used to renew the session key.

Asymmetric cryptography is used in varying degrees in all scenarios. This cryptography requires both parties exchanging data to ensure that each party has its own asymmetrical key. The procedure for determining the session key for these parties requires exchanging their public keys. To ensure the secure exchange of public keys between the parties, the so-called “*third party*” of trust [4], known as the Certification Authority, is needed. The described solutions are known and widely used but require that each node of the network have access to the network in which the Certification Authority is reachable. In addition, the algorithms used require relatively large memory resources for stored keys; the device must have adequate computing power and a sufficiently efficient energy source. These requirements are easy to meet by stationary nodes, but are a big challenge for usually mobile, using wireless links and battery-powered nodes of sensor network, which currently constitute a very large population of IoT network nodes [5,6].

Looking at the development of the network, it can be seen that, despite the availability and proven universal solutions for data exchange in the network and for data protection, various organizations create clusters of network nodes in which specialized security rules adapted to the organization’s requirements apply [7,8]. This approach is designed to protect data exchanged within the organization against unauthorized activities from outside the organization.

Such an organization can be a large corporation, elements of the state’s critical infrastructure, the armed forces, but it can also be one of the IoT applications e.g., a distributed system of measuring air pollution, a system for monitoring the consumption of electricity by both private and public consumers, a system for monitoring public transport vehicles in a smart city, clusters of sensor nodes performing specific tasks, and also systems classified as critical infrastructure, e.g., systems used by the police or army. Particularly in the event of a crisis, the highest demand is for current data, which can often come from a variety of sources that cannot always be trusted. Crisis situations will force the secure exchange of sensitive data between well-protected elements of critical infrastructure through little trusted ICT links. An example of such cooperation can be the integration of commercial and civil Internet of Things with military C2 (Command and Control) and logistics systems [9,10].

Certainly, any solution used to integrate such different systems, technologies, and security rules will require a source of symmetric cryptographic keys with high entropy, which will be trusted by all parties. This article describes the concept of a system for generating and renewing symmetric cryptographic keys called the Key Generating and Renewing (KGR) system for customers who have only limited resources in terms of memory, computing power, and energy supply, as well as for customers who do not have such limitations. The main tasks of the system are the secure delivery of a new key and renewal of an expired symmetric key for the parties to exchange data. The inspiration for the solution was the need for secure and authorized data exchange ensuring Federated Interoperability of Military Command and Control and IoT systems. In such a federation, individual groups of network nodes may belong to different national armed forces, within which cryptographic security is inherently used, but not shared with other members of the federation. The main contributions of this paper are presented as follows:

- Description of the concept of the KGR system intended for the safe distribution and renewal of cryptographic keys for sensor nodes, which are representatives of sensor node clusters formulating the security domain of sensor nodes [7,8]. These keys are designed to protect secure data exchange between domains. Within each secure domain, independent of the KGR system, trust structures are built and implemented mechanisms of data exchange protection and data storage protection.
- A method of building trust between KGR system nodes and protecting the resources of each node of the KGR system.
- A detailed description of the procedures and protocols for data exchange intended for: initiating the KGR system, preparing nodes for work, and registering the nodes in the KGR system as well as generating, renewing, and distributing keys.
- Description of how to use the Trusted Platform Module (TPM) v2.0 hardware modules to support procedures for creating trust, protection of sensor node resources and securing data exchange in the KGR system.
- Description of how to use the Message Queuing Telemetry Transport (MQTT) service to securely distribute data (including cryptographic keys) between KGR system nodes.
- Evaluation of the solution resistance to the most common attacks on the sensor node network.

The rest of the article is organized as follows. Related work is the content of Section 2. Section 3 presents the concept of KGR system operation and data structures used by system nodes. Section 4 is devoted to a detailed description of the procedures necessary for the proper functioning of the KGR system. The security evaluation of the proposed system is presented in Section 5. The future work is the content of Section 6.

## 2. Related Work

In the era of the rapidly growing Internet of Things, a very big challenge is to ensure confidentiality, integrity, and accessibility, which are the main attributes of system security. A large degree of difficulty is compounded by the following facts: IoT nodes are very often mobile, have limited memory resources, computing power and power sources, limited communication link range, are usually very numerous, and operate in difficult environmental conditions and usually in an unattended environment. Security mechanisms based on cryptographic solutions are used to build the trust of cooperating parties and meet security requirements. The implementation of these mechanisms further increases the requirements in terms of memory size and computing power. A critical element of such solutions is the generation and renewal of cryptographic keys as well as the distribution of these keys to nodes exchanging the data. Well-known solutions for building trust and distribution of cryptographic keys can be used here, e.g., Certification Authorities, but in the case of devices with limited capabilities this approach will be effective in few cases.

Attempts to find a compromise between the requirements described above most often resulted in an approach in which a large network of sensor nodes was divided into groups of cooperating nodes for which group key management (GKM) was used as a key distribution mechanism. Keys distributed using GKM are usually shared by nodes forming one group. A very extensive analysis of GKM systems can be found in [11]. Dammak et al. in [11] analyze the properties of GKM systems due to their applications for: wireless body area networks (WBAN) [12], wireless sensor networks (WSN) [13,14,15], cloud computing [16], wireless IPv6 networks [17], and IoT [18,19,20]. They compare solutions for individual groups of GKM applications, taking into account many attributes, the most important of which are: key distribution schemes (i.e., centralized, decentralized, distributed [21]), cryptography type used (symmetric, asymmetric, polynomial, Attribute Based Encryption), forward and backward secrecy (the shared key must be updated when a new node joins the group or some node leaves the group), mutual key independence, existence of single point of failure, and scalability.

The conclusions of this analysis can be described in the following. The analyzed solutions do not fully solve the problem of having to renew the key if a node joins/leaves the group. In most cases, symmetric cryptography algorithms are used. The performance of solutions quickly decreases when the number of nodes is large, or the membership of cooperating groups changes frequently. The scalability of the solutions is very limited. Against the background of these conclusions, in [11] is presented the Decentralized Lightweight Group Key Management architecture for Access Control in the IoT environment (DLGKM-AC). The solution is flexible and well scalable in a dynamic IoT environment, uses a decentralized key distribution scheme, and does not cause a heavy load on the GKM system in the event of changes in the membership of nodes to the group. Another interesting GKM solution is proposed by Yao et al.’s key distribution scheme based on Logical Key Hierarchy [22]. This solution presents a method of building a key tree and a method of storing keys that reduces the computational and memory load of individual sensor nodes. The system also supports the renewal of cryptographic keys.

Another innovative approach to key generation and distribution is the use of the block chain mechanism. This approach can be used in mobile sensor node networks. However, in these networks, in addition to numerous sensor nodes implemented on constrained devices (L-sensors), there must be, although much less numerous, nodes (H-sensors) with large data storage, and computational and communication capabilities. H-sensors nodes form a consensus network and act as heads for node clusters that will include L-sensors nodes. An example of such a solution was presented by Tian et al. in [23].

## 3. The Concept of the Cryptographic Keys Generating and Renewing (KGR) System

### 3.1. The Idea of KGR System Operation

The clients of the KGR system will be mobile sensor node networks. Each sensor node network forms one secure sensor node domain [7,8] in which data exchange is cryptographically secured. Security mechanisms implemented in this domain also ensure the generation and distribution of cryptographic keys inside the domain and cryptographic protection of the resources of each node.

One security domain of sensor nodes is formed by sensor nodes that are registered in the domain. Each sensor node, in addition to performing the normal sensor and/or actuator tasks, plays the role of Master or Replica in the domain. At a given moment, exactly one sensor node in a domain is an authority in the domain. This node acts as a Master. Its resources store up-to-date descriptions of the domain and descriptions of all sensor nodes of the domain as well as data necessary for domain node authentication.

The remaining sensor nodes of the domain play a Replica role. These nodes in their resources store a copy of the safe domain description obtained from the Master node. In the event of a failure of the sensor node acting as the Master, a special procedure for selecting a new Master node from among correctly functioning Replica nodes is launched.

**Figure 1:**
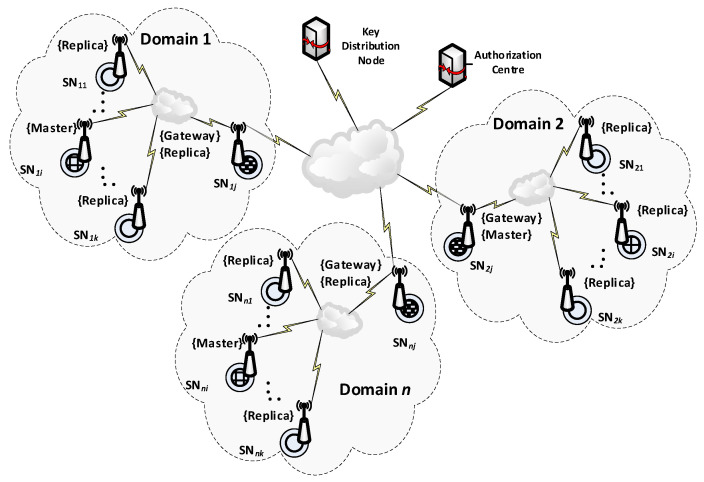
The way various domains cooperate with the key distribution node.

One of the nodes of each domain (it may be either a Master or Replica sensor node) is designated for data exchange between such domains and acts as a Gateway for the domain. This node is a sink node for data originating from domain nodes, but also an emitter of data originating outside the domain for domain nodes. The gateway node is responsible for secure data exchange between domains and is the representative of the domain for the KGR system. For the KGR system, out of all security domain nodes, only the sensor node is important, which at the given moment acts as a Gateway in the security domain of the sensor nodes. In the KGR system, the source of cryptographic keys to protect data transmission will be a separate node (Key Distribution Node) to which the gateway nodes will have access. The way the domains cooperate with the key distribution node is shown in Figure 1.

There will be three types of nodes in the KGR system:

- KS node (Key Server)—the node that is equivalent to the Key Distribution Node in Figure 1 and will be the source of the cryptographic keys;
- N1, N2, … N*_k_* nodes—the representative for domains for whom symmetrical keys will be created;
- AC node—Authorization Centre—a server for two services:
  ⚬ The service for managing the resources of the KS node, and for adding new identifiers for the authorized N nodes in the resources of the KS node;
  ⚬ The service to prepare N*_k_* nodes for work.

### 3.2. The Method of Data Exchange in the KGR System

During one key generation operation, the KS node will prepare two symmetrical keys, NNSK and NNSKsign, which will be used by a pair of nodes—N*_m_* and N*_n_*. The NNSK key (*Node to Node Security Key*) will be used to encrypt data sent between the N*_m_* and N*_n_* nodes, and the NNSKsign (*Node to Node Security Key for signing*) key will be used to prepare the HMAC for transmitted data. The sequence of activities carried out during one key generation operation can be described as follows:

- Node N*_m_* sends to KS node a request to generate a pair of symmetric keys for N*_m_* and N*_n_* nodes;
- Node KS generate NNSK and NNSKsign keys and temporarily stores them;
- KS node sends the keys to N*_m_* and N*_n_* nodes;
- Node N*_n_* via KS node sends confirmation of key receipt to N*_m_* node;
- After sending the confirmation, KS node removes the NNSK and NNSKsign keys from its resources.

**Figure 2:**
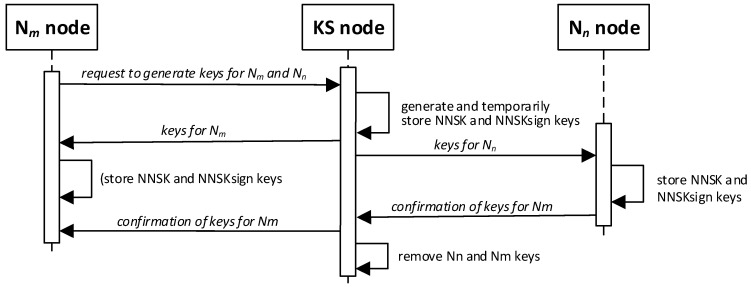
Sequence diagram of key generation for node pair N*_m_* and N*_n_*.

**Figure 3:**
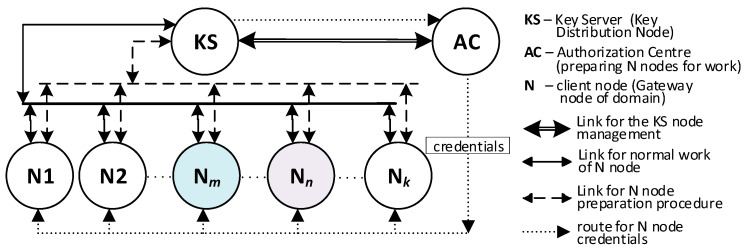
The method of data exchange in the Key Generating and Renewing (KGR) system.

The sequence diagram for these activities is shown in Figure 2. The method of data exchange in the KGR system is shown in Figure 3.

### 3.3. Proposed KGR System for Mobile IoT Network

Considering the observations mentioned in the introduction and the fact that one of the parties to the data exchange may be a mobile IoT network node that has limited memory, and computational and energy resources, it was assumed that the designed system should meet the following assumptions:

- The KGR system is a source of symmetric cryptographic keys generated using a high entropy random number generator (e.g., a quantum random number generator).
- The KGR system is available on the Internet.
- The KGR system only handles requests from authorized clients.
- Data on authorized clients are provided to the KS node by the administrator of the KGR system.
- On behalf of each client of the KGR system, there is one node that is the client’s representative.
- The client representative can be implemented in hardware, but there can also be a software component installed on a computer network node that has access to the KGR system.
- Representatives of two clients applying for a new pair of shared cryptographic keys are the only nodes with which the KGR system exchanges data related to obtaining a common pair of symmetric keys only for these clients.
- The MQTT protocol will be used to distribute cryptographic keys.
- Sensitive data stored in the resources of each node are cryptographically protected.
- Data transmission between nodes is protected by cryptography.
- Each node uses the local trust structure.
- Each node must be registered in the system before it can start normal working—registered nodes before they begin their activities must be authenticated.
- The security mechanisms offered by the Trusted Platform Module (TPM) will be used to secure data resources and secure data exchange between elements of the KGR system and its clients. TPM is an implementation of a standard developed by the Trusted Computing Group [24,25]. This module is designed to hardware support the cryptographic procedures and protocols that can be used for securing data [2,26].

### 3.4. Key Exchange Domain (KED) Structure

**Figure 4:**
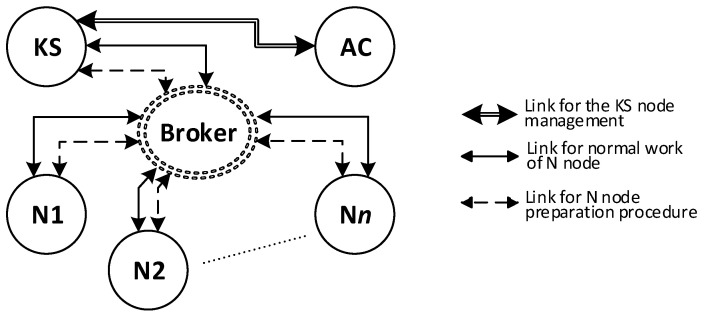
Structure of a Key Exchange Domain.

Nodes of the KGR system will form the Key Exchange Domain (KED). There will be one KS node, one AC node and many N-type nodes in the KED domain. The Broker node will act as a data exchange intermediary between these nodes. The KED domain structure is shown in Figure 4.

One of the important elements that determines the correct use of the MQTT service is determining the content of the special string, which is called “*topic*”. The task of the Broker node is to forward data published by one node with a given “*topic*” to the node that subscribes to messages with such “*topic*”. The presented solution assumes that the content of individual “*topic*” strings will be randomly generated and known only for a pair of nodes that will exchange data with each other. In the further part of the study, these character strings will be marked as TOPIC*n*, where *n* = 0, 1, 2, 3, 4. The description of the purpose of each topic is presented in the Table 1.

Depending on the demand and the available Trusted Platform Module (TPM) model, the N-type node can be implemented as:

- An autonomous device equipped with a hardware TPM, which implements all the features defined in the specification TCG [24];
- An autonomous device equipped with a hardware TPM, which does not support the hardware features encryption/decryption, these modules do not support the encrypt/decrypt function (an example would be two hardware implementations, i.e., LetsTrust TPM 2.0 and Infineon Iridium SLx 9670 available on the market, according to their documentation, they do not support encryption/decryption functions);
- An autonomous device equipped with a software simulator TPM, which implements all the features defined in the specification TCG [24];
- A program module that does not use the TPM module but provides the generation and renewal of cryptographic keys for the system of which it is representative.

The KGR system concept presented in the work is prepared for the case in which the TPM hardware modules used do not have the symmetric encryption/decryption function implemented. Therefore, symmetric encryption/decryption must be performed by software. An additional adverse effect of the lack of hardware encryption/decryption function is also the inability to use the “Key Derivation Function” function offered by the TPM module. The lack of this function means that in the NVRAM memory of the TPM module, instead of one “seed” string, two keys will have to be stored, which will be a specific pair, i.e., accordingly, a symmetric key for encrypting/decrypting data and a key for determining HMAC for these data.

### 3.5. The Concept of the Nodes Protection

It was assumed that KS node and N nodes would use their local trust structure. This structure will be used to provide protection for sensitive node data, build trust relationships between KED domain nodes, and to protect data exchange between KED domain nodes. For this reason, it is recommended that each of the KED domain nodes be equipped with the TPM v2.0 module. For this purpose, a hardware TPM v.2.0 was planned, implemented on the extension board attached to the sensor node board (e.g., Raspberry Pi).

TPM is the international standard for a secure crypto processor designed to secure hardware through integrated cryptographic keys. It can be implemented in software or in hardware. TPM typically implements multiple cryptographic algorithms (e.g., SHA- (256, 384, 512), HMAC, RSA (2048, 3072, 16384), ECC (256, 384, 521), and AES (128, 256)) and crypto primitives (e.g., a random number generator, key generation, a public-key cryptographic algorithm, a cryptographic hash function, a mask generation function, digital signature generation and verification, ECC-based Direct Anonymous Attestation, and symmetric-key algorithms). TPM enables the building of several hierarchies of cryptographic keys (platform, storage, and endorsement hierarchies) that can be used to build a local trust structure on each TPM-equipped node. The key hierarchy is built from asymmetric keys. At the top of each hierarchy, there is an asymmetric key, which for a given TPM chip is randomly generated only once, and its private part never leaves the TPM chip and is not readable. With this key, the next key in the hierarchy is secured, etc. Any key that is secured using the key hierarchy is not shared outside the TPM chip in an explicit form. All cryptographic operations with the use of these keys are performed inside the TPM chip. In the TPM module NVRAM, it is possible to safely store sensitive system data (e.g., cryptographic keys). The PCR (Platform Configuration Register) of TPM makes it possible to build a system for detecting unauthorized hardware and software modifications of the resources of the node where the TPM is installed.

Because KS and AC nodes will be nodes of the Internet and will not have restrictions in terms of memory size, computing power, and energy, the possibilities of the Certification Authority will be used to build trust relationships between them.

The local trust structure will be constructed based on one of the hierarchies offered by the TPM v.2.0 module (i.e., storage hierarchy) built from asymmetric keys (e.g., RSA2048 or ECC256). It is expected that the Storage Root Key (SRK) will be at the top of the hierarchy, and the next key will be the asymmetric node key ANK (Asymmetric Node Key) for signing other keys that will be used by the node. For the KS node, the second child of the SRK key will be the KEDK (Key Exchange Domain Key), which will be needed to create the trust relationship between the KS node and the other N-type nodes.

#### 3.5.1. Characteristics of the KS Node

The KS node plays the most important role in the KGR system and is responsible for generating and renewing symmetric keys requested by N nodes and their safe distribution to the node requesting the key and its partner. The data that are necessary to perform these tasks can be divided into the following groups:

- Local trust structure of the KS node;
- The node’s own data;
- Data for storing node descriptions (KED domain node data) that can use the services offered by the KS node;
- Temporarily stored data that contain cryptographic material created for nodes that are in the process of generating or renewing a symmetric key for those nodes.

**Figure 5:**
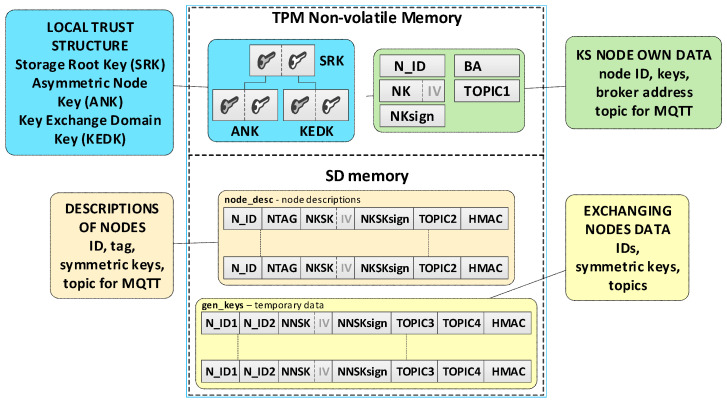
Data stored on the KS node.

It was assumed that this KS node’s own data and its local trust structure will be stored in the TPM NVRAM installed on the node. KED domain description data and sensitive temporary data will be stored in the node’s SD memory. All mentioned data will be cryptographically secured using TPM mechanisms. The data stored on the KS node are shown in Figure 5.

Individual data of the KS node include:

- Asymmetric keys SRK, ANK, and KEDK which create local trust structure;
- N_ID—sensor node identifier in the domain;
- Keys:
  ⚬ NK (Node Key)—symmetric key for encrypting the data stored in local SD memory (the key is used only internally by this node);
  ⚬ NKsign—key for determining HMAC for data encrypted with the NK key (the key is known only to this node);
    - BA (Broker Address)—MQTT broker IP address;
    - TOPIC1—topic subscribed by KS in the MQTT service used to initiate the generation and renewal of session keys by N nodes, common to all registered N nodes (10-byte random string generated by the KS node).

Data stored in SD memory of KS node:

1. File **node_desc**—stores data of KED domain nodes that can use the services offered by the KS node. One record in the file includes a description of one node. If only the N_ID and NTAG fields of a given record are filled, then the node status is “*authorized to register*”. When all the description fields of the node are filled, the node is “*registered*”. Each node description (except the N_ID field) is encrypted with the NK key. After encryption, the HMAC value is generated for the entire record using the NKsign key. Description of the record fields is as the following:
  - N_ID (Node ID)—identifier of the node authorized to register in the domain (random number 4 bytes long—field generated during the procedure of adding a new authorized node to the domain description);
  - NTAG (Node Tag)—tag for the node; SHA256 hash from the concatenation of the N_ID field, the public part of the KEDK key, and a double-byte field containing the node description entry number in the node_desc file (field generated during the procedure of adding a new authorized node to the domain description);
  - NKSK (Node to Key server Security Key)—a symmetrical key for securing data exchange between a registered node and a KS node (field created during the node registration procedure);
  - NKSKsign—key for determining HMAC for data encrypted with the NKSK key (the key is only known for the node and KS node);
  - TOPIC2—topic subscribed by the N node in the MQTT service used to exchange data related to generating/renewing session key for N node (10-byte random string generated by the N node);
  - HMAC—HMAC value for the entire record, which is generated using the NKsign key.
2. File **gen_keys**—temporarily stores the generated keys, i.e., from the moment they are generated until the confirmation of receipt of these keys by the nodes for which these keys were generated. Each record of the file (except the N_ID1 and N_ID2 fields) is encrypted with the NK key. After encryption, the HMAC value is generated for the entire record using the NKsign key. Description of the record fields is as follows:
  - N_ID1—identifier of the node that sent the request to generate/renew the key for the pair of nodes N_ID1 and N_ID2;
  - N_ID2—identifier of the second node in the pair for which the key was generated/renewed;
  - TOPIC3—topic subscribed by N_ID1 in the MQTT service to receive data from the N_ID2 node;
  - TOPIC4—topic subscribed by N_ID2 in the MQTT service to receive data from the N_ID1 node.

Due to the fact that the KS node plays the most important role in the system and must be available at all times to system customers, it should be implemented rather as a stationary device that is powered from a constant power source and connected to an efficient Internet connection. The node should be properly configured from a security point of view and protected using tools such as a firewall, IDS/IPS systems, antivirus software, etc. Considering that clients may be different nodes of N-type, which will not necessarily have a commonly used Ethernet or Wi-Fi connection in the Internet, and most often will use other communication technologies specific to IoT networks, the KS node should be equipped with an additional device which will act as a gateway for other communication technologies, e.g., LoRa, Xbee, ZigBee, BLE etc.

#### 3.5.2. Characteristics of N-Type Nodes

The clients of the cryptographic key generation and renewal service are N nodes. Each such node will store in its resources the necessary data to establish a connection and secure data exchange with the KS node, as well as the data necessary for secure data exchange with other N nodes. The data that are necessary to perform these tasks can be divided into the following groups:

- Local trust structure of N node;
- Node’s own data;
- Data on current session keys to secure data exchange with other N-type nodes.

**Figure 6:**
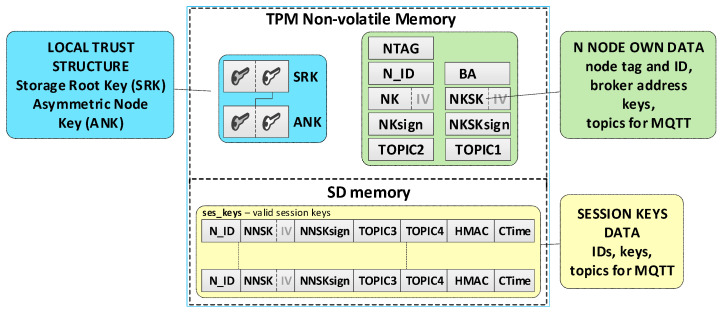
Data stored on the N node.

It was assumed that this N node’s own data and its local trust structure would be stored in the node’s NVRAM of the TPM. Data about the current session keys will be stored in the node’s SD memory. All mentioned data will be cryptographically secured using TPM mechanisms. These are the secure storage of data in the NVRAM of the TPM, the use of the HMAC hash generation function for each record in the SD memory, and the symmetric encryption/decryption feature that uses keys that are securely stored and shared by previously created hierarchies of keys. Data stored on the KS node are shown in Figure 6.

Individual data of the N node include:

- Asymmetric keys SRK and ANK which create a local trust structure.
- NTAG (Node Tag)—tag for the node; obtained from the AC node during the procedure of preparing the node for work in the KED domain.
- N_ID—sensor node identifier; obtained from the AC node during the procedure of preparing the node for work in the KED domain.
- BA (Broker Address)—MQTT broker IP address.
- keys:
  ⚬ NK (Node Key)—symmetric key for encrypting the data stored in local SD memory (the key is used only internally by this node);
  ⚬ NKsign—key for determining HMAC for data encrypted with the NK key (the key is known only to this node);
  ⚬ NKSK (Node to Key server Security Key)—a symmetrical key for securing data exchange between a registered node and a KS node (known only for the given node and KS node); created during the procedure of registering the node in the KED domain;
  ⚬ NKSKsign—key for determining HMAC for data encrypted with the NKSK key (known only for the given node and KS node); created during the procedure of registering the node in the KED domain.
- TOPIC1—topic subscribed by KS in the MQTT service used to initiate the generation and renewal of session keys by N nodes, common to all registered N nodes.
- TOPIC2—topic subscribed by node N in the MQTT service for exchanging data with the node KS related to generating/renewing session key.

Data stored in SD memory of N node:

1. File **ses_keys**—stores data about valid symmetrical session keys to secure data exchange between a given node and a node with identifier N_ID. One record in the file includes a description of one session key. If the CTime field is zeroed, it means that the session key has not been generated yet or expired. Each session key description (except the N_ID and CTime fields) is encrypted with the NK key. After encryption, the HMAC value is generated for the entire record using the NKsign key. Description of the record fields is as follows:
  - N_ID (*Node ID*)—identifier of the target node for which the valid session key is NNSK;
  - NNSK (Node to Node Security Key)—a symmetrical key for securing data exchange between a given node and a node with the identifier N_ID (known only for the given node and node with the identifier N_ID);
  - NNSKsign—key for determining HMAC for data encrypted with the NNSK key (known only for the given node and node with the identifier N_ID);
  - TOPIC3—topic subscribed by the local node to receive data from the N_ID node;
  - TOPIC4—topic subscribed by node N_ID to receive data from the local node;
  - CTime (Creation Time of the key)—time stamp of the moment of obtaining the session key.

When developing the concept of the KGR system, small requirements were specified for N-type nodes. These nodes, to be clients of the KGR system, should have a communication link ensuring cooperation with the KS node. Regarding N nodes, the following assumptions were made [27]:

- Node N is a class 1 (RAM << 10 KB and Flash << 100 KB) or class 2 device (RAM ~ 10 KB and Flash ~ 100 KB) constrained device;
- Node N is powered by a class E1 energy source (i.e., “*Period energy-limited*”, for example battery that is periodically recharged or replaced) or E2 (“*Lifetime energy-limited*”, for example nonreplaceable primary battery).

This means that the N node can be a mobile device using a wireless link, has limited memory resources, limited computing power, and is powered from a limited capacity energy source.

#### 3.5.3. Characteristics of the AC Node

It was assumed that the KS node would only support authorized N-type nodes. To accomplish this goal, it is necessary to provide the KS and N-type nodes with relevant data. The KS node will require the information on N-type nodes that will be entitled to cooperate with the KS node. For N-type nodes, you will need the credentials that will enable the initiation of cooperation between the given N node and the KS node. These activities define the main tasks of the AC node in the KGR system.

Due to the fact that the KGR system is designed to support systems that are very diverse in many respects, e.g., in terms of purpose, technology used, information processing method, method of protecting system resources, classification of processed data, organizational and national affiliation, etc., it is very difficult to define a uniform way of cooperation between such systems. Therefore, it was assumed that the process of initiating such cooperation must be based on organizational procedures that will be supported by the AC node. It is expected that AC node resources will store data that reflect these organizational processes. The specification of these processes is not the content of this study.

From the security point of view, the critical moment of initiating cooperation between systems will be the transfer of authentication data to N-type nodes (because they are constrained devices class 1 or 2), which will not be able to fully use the mechanisms of secure data exchange currently used in the Internet. These activities should be performed in a controlled and safe environment. Considering the above observations, it was assumed that the AC node will perform the following tasks:

(a) Securely forwarding to the KS node a list of N nodes that will be entitled to use the service of the KGR system and obtaining from the KS node the data necessary to initiate cooperation of each of these nodes with the KS node. This data will include the identifier (N_ID) for N node and the tag (NTAG) for this node;
(b) Securely forwarding the credentials prepared by the KS node to authorized N node. These data include the N_ID for N node and the NTAG for this node;
(c) Secure transfer to the authorized N node of a list containing the identifiers of other N-type nodes with which the given node will be able to cooperate.

The manner of implementing the above tasks is not the content of the study. It is expected that the task described in item (a) will be able to be performed through the internet application using the HTTPS protocol or using the MQTT service. For the purposes of the study, it was assumed that the data that are the result of the task described in subsection (b) will be transferred via a text file containing one record, which will contain the authentication data for the N node (i.e., N_ID, NTAG and BA). Data that are the result of the task described in subsection (c) will also be transferred via a text file that will contain a list of identifiers of other N-type nodes with which the given node will be able to cooperate.

## 4. Procedures in the Key Exchange Domain

Building a secure system requires designing security solutions for the software and hardware configuration of all system components. An important element of these solutions is secure procedures for creating such a system and secure procedures for using such a system and decommissioning it. The following procedures are foreseen for the KGR system:

1. The procedure for starting the Broker node;
2. **The procedure for initiating the KS node**;
3. The procedure for preparing the credentials for the N node;
4. **The procedure for initiating N node**;
5. **The procedure for registration N node in the KED domain**;
6. Procedure for forwarding the list of authorized nodes to cooperation.
7. **The procedure for generating session keys** consists of three stages:
  (a) Requesting the session key;
  (b) Providing the session key to the destination node;
  (c) Confirmation of the delivery of the session key to the destination node.
8. **Procedure for secure data exchange between nodes**.
9. The procedure for renewing the session key consists of three stages:
  (a) Session key renewal request—includes the process of notifying the other party that the procedure has been initiated;
  (b) Providing a renewed session key to the destination node;
  (c) Confirmation of the delivery of the renewed session key to the destination node.

The next part of the study contains descriptions of the procedures. In the above list, the procedure names that are described in detail, are marked in bold.

### 4.1. The Procedure for Starting the Broker Node

The Broker node acts as an intermediary in the exchange of data between nodes of the KGR system. Activities related to launching the MQTT service should be performed first when initiating the KGR system. It was assumed that the service must be configured so that data exchange between KGR system nodes is secured using the TLS (Transport Layer Security) mechanism. In addition, the network node on which the MQTT service operates should be properly configured from a security point of view in accordance with good practices in this field. Actions connected with launching the MQTT service are not the content of this study.

### 4.2. The Procedure for Initiating KS Node

**Figure 7:**
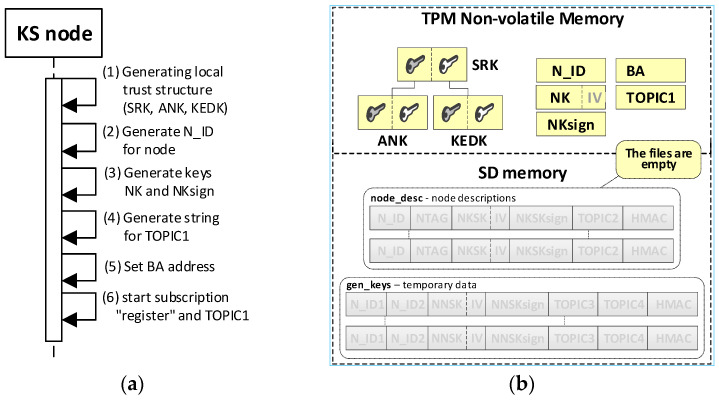
The sequence diagram for the KS node initialization procedure (**a**) and the data stored on the KS node after the initialization procedure (**b**).

The KS node initialization procedure is the first step that must be performed to start the KGR system. The purpose of this procedure is to create a local trust structure for the KS node (including asymmetric keys SRK, ANK, and KEDK) and create individual data for the node. These data include: node identifier N_ID, symmetric key NK and the accompanying NKsign key for securing data stored in the node’s SD memory, MQTT broker address, and topic TOPIC1 which will be subscribed by the KS node in the MQTT service to generate or renew the session keys for N nodes. The last step of the procedure is to start subscription to the topic TOPIC0 and TOPIC1, which establishes the readiness of the KS node to work. The sequence diagram for the KS node initialization procedure and the data stored on the KS node after the procedure are shown in Figure 7 (the updated data are highlighted in yellow).

### 4.3. The Procedure for Preparing the Credentials for N Nodes

**Figure 8:**
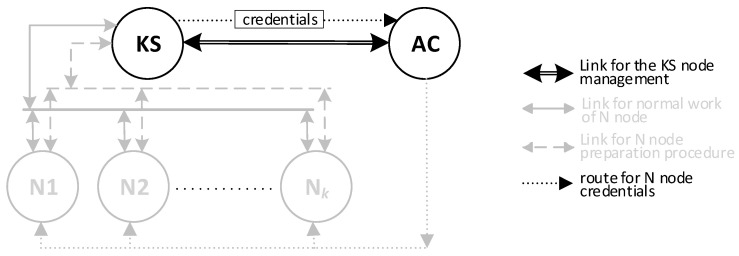
The way the AC node and the KS node work together during the procedure for preparing the credentials for the given N node.

According to suggestions in the description of the AC node (Section 3.5.3), this procedure is not described here in detail. The AC node sends request to the KS node to prepare credentials for the given N node. The KS node generates the credentials and sends them to the AC node. The way the two nodes work together during this procedure is shown in Figure 8.

**Figure 9:**
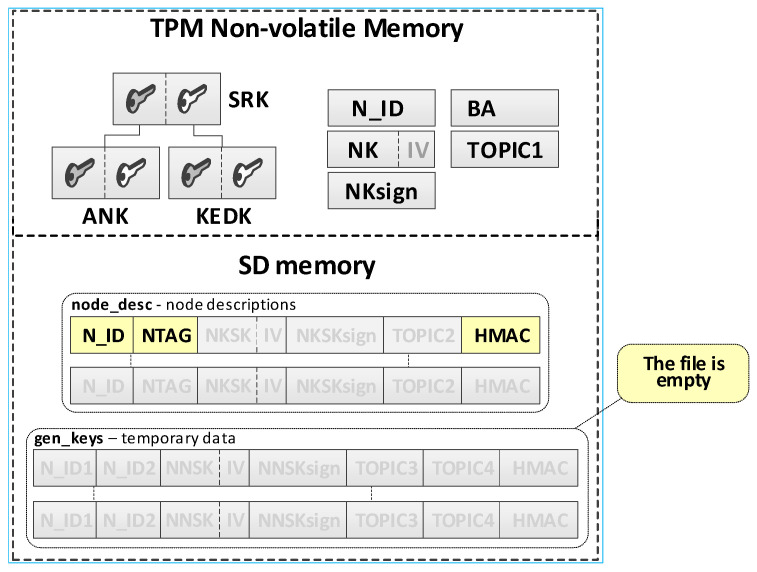
An example of data stored on the KS node after adding the description of the first N node.

The result of this procedure is the appearance of a new entry in the **node_desc** file stored in the resources of the KS node and a text file containing the credentials intended for given N node that have just been added to the resources of the KS node. An example of data stored on the KS node after the procedure is shown in Figure 9. (the updated data after adding the first node are highlighted in yellow).

### 4.4. The Procedure for Initiating the N Node

**Figure 10:**
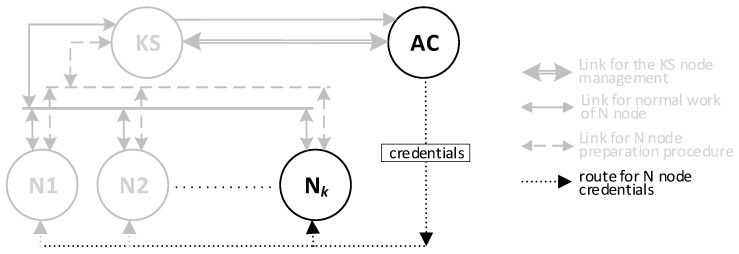
The way the AC node and the N node work together during the procedure for initiating the N node.

**Figure 11:**
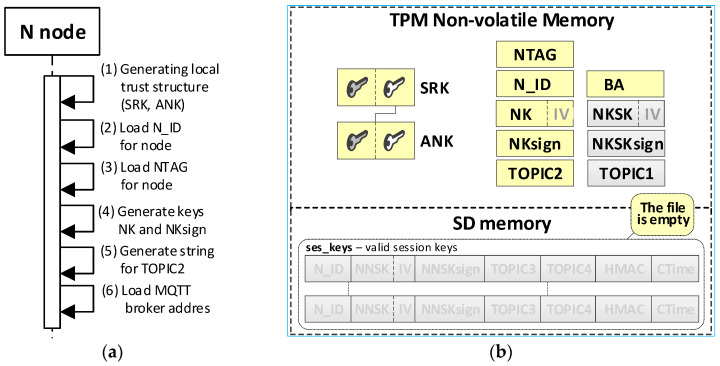
The sequence diagram for the N node initialization procedure (**a**) and the data stored on the KS node after the initialization procedure (**b**).

The procedure for initiating the given N node aims to create a local trust structure for the N node (including asymmetric keys SRK and ANK), load the credentials obtained from the AC node, and create individual data for the node. Loaded data include: node tag NTAG and node identifier N_ID. Generated data include: symmetric key NK and the accompanying NKsign key for securing data stored in the node’s SD memory, and topic TOPIC2, which will be subscribed by the given N node in the MQTT service during the procedure for registration the N node in the KED domain and the procedure of generating or renewing the session keys for the N node. In the last step, the IP address of the MQTT broker is loaded to the BA field. The way the AC node and the N node work together during this procedure is shown in Figure 10. The sequence diagram for the procedure and the data stored on the N node after the procedure are shown in Figure 11 (the updated data after the initialization procedure for the N node are highlighted in yellow).

### 4.5. The Procedure for Registration N Node in KED Domain

This procedure is intended to register the N node in the resources of the KS node and generate and transfer to the registered node the NKSK and NKSKSign keys to secure future data exchange between the given N node and KS node. Data from the N_ID, NTAG fields of the registered node, which this node obtained from the AC node in the node initialization procedure, and TOPIC2 field are encrypted using the contents of the NTAG field (the contents of this field will be used as a “*one time password*”). HMAC is determined for all sent data also using NTAG, and then the data are sent to the KS node. The KS node checks the authorization of the received request. If the KS node detects any abnormalities in the request, it ignores the request. These abnormalities include the following situations: in the KS resource, no description of the node that issued the request, an invalid NTAG, or an invalid HMAC hash.

**Figure 12:**
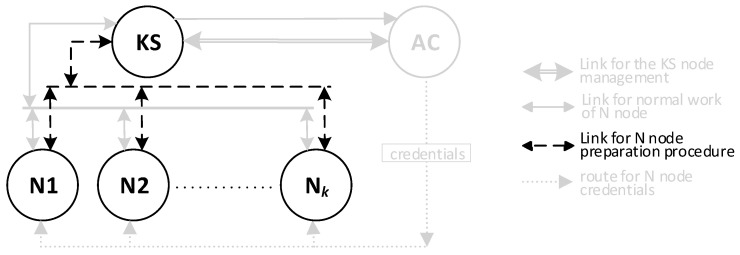
The way the KS node and N node work together during the procedure for registration of the N node.

**Figure 13:**
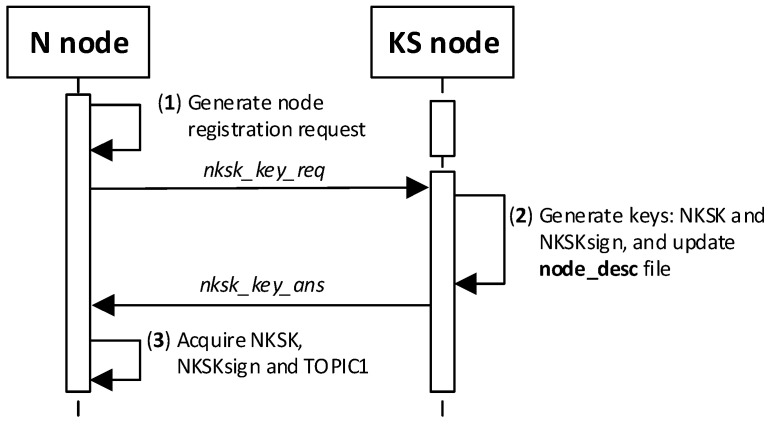
The sequence diagram for N node registration procedure.

In the next step, the KS node generates NKSK and NKSKsign keys for the registered node, completes the description of this node in the local file node_desc, and in response sends the generated keys supplemented with the field TOPIC1 to the registered node. The contents of the NTAG field of the node being registered is used to protect the response. The MQTT service is used during the registration procedure. The way the KS node and the N node work together during this procedure is shown in Figure 12. The sequence diagram of the procedure for the registration of the node in the domain is shown in Figure 13.

**Figure 14:**
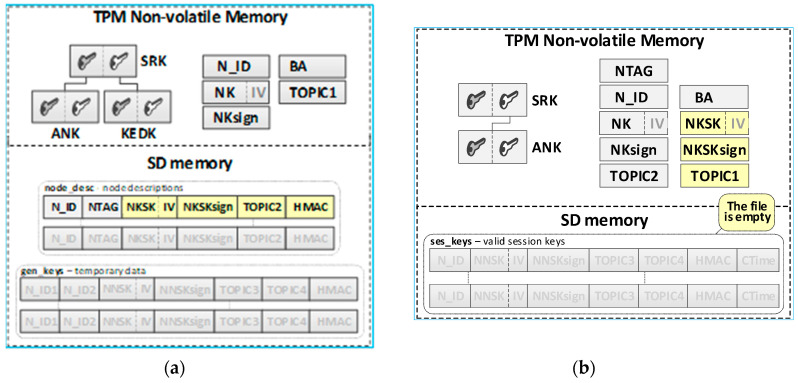
The sequence diagram for the N node initialization procedure (**a**) and the data stored on the KS node after the initialization procedure (**b**).

The result of the node registration procedure in the key exchange domain is the update of data in the KS node resources and the resources of the registered node. The contents of the KS node and the first registered node in the domain are shown in Figure 14 (the updated data after the registration procedure are highlighted in yellow).

**Figure 15:**
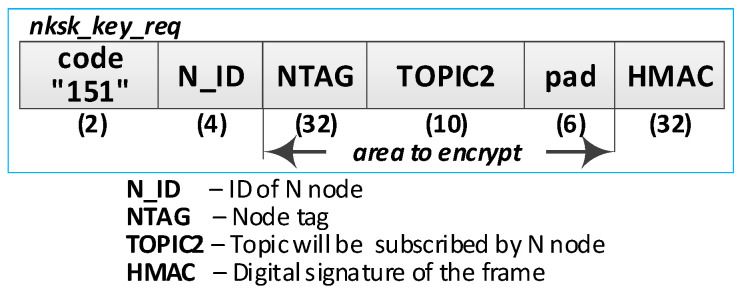
The node registration request frame.

**Figure 16:**
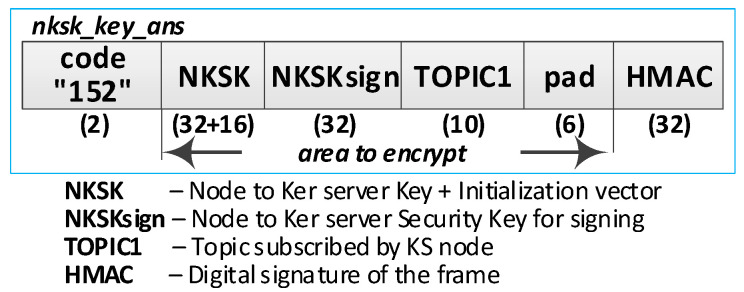
The confirmation frame for node registration.

Detailed descriptions of the most important steps of the procedure (in Figure 13, the numbers in parentheses (e.g., (2)) preceding the descriptions indicate the numbers of the individual steps) are as follows:

**(1)**  **Generate node registration request**—a node registration request sent to the KS node is also a request to send the keys NKSK and NKSKsign, and string TOPIC1 back to the N node. Conduct the following:
  - Prepare the ***nksk_key_req*** packet containing the following data: N_ID, NTAG, and TOPIC2 of the registered N node (Figure 15). The packet is encrypted using the string from NTAG field, and NTAG is also used to determine the HMAC hash;
  - Publish a ***nksk_key_req*** packet using the topic TOPIC0.
**(2)**  Generate keys: NKSK and NKSKsign and update the N node description in the **node_desc** file. Conduct the following:
  - On the KS node, decrypt the data from the ***nksk_key_req*** packet using the NTAG from the description of the N_ID node and verify the correctness of the NTAG field from the packet. If it is not correct, stop the procedure.
  - On the KS node, generate the keys NKSK with the initialization vector and NKSKsign, prepare the N node description, and then encrypt this description using the NK and the IV vector of the KS node, determine the HMAC using NKsign, and update the N node description in local file **node_desc**. The fields of the description should have the following values:
    N_ID = the content remains unchanged (the field is not encrypted);
    NTAG = the content remains unchanged;
    NKSK and IV = value generated by the KS node;
    NKSKsign = value generated by the KS node;
    TOPIC2 = TOPIC2 gathered from the nksk_key_req packet.
  - Publish a confirmation of registration to node N (***nksk_key_ans*** packet in Figure 16) using topic TOPIC2 of registered node. The confirmation contains NKSK, IV, NKSKsign, TOPIC1. The fields are encrypted using the NTAG of the registered node, and for the encrypted blob, the HMAC is determined also using NTAG of the registered node.
**(3)**  **Acquire NKSK, NKSKsign, and TOPIC1**. Conduct the following:
  - On the N node, decrypt received the nksk_key_ans packet using NTAG, save the received data in NVRAM of TPM. The data should have the following values:
    NKSK and IV = NKSK gathered from the nksk_key_ans packet;
    NKSKsign = NKSKsign gathered from the nksk_key_ans packet;
    TOPIC1 = TOPIC1 gathered from the nksk_key_req packet.

The contents of the N node after registration in the domain are shown on the right side of the Figure 14 (the updated data after the registration procedure are highlighted in yellow).

**Figure 17:**
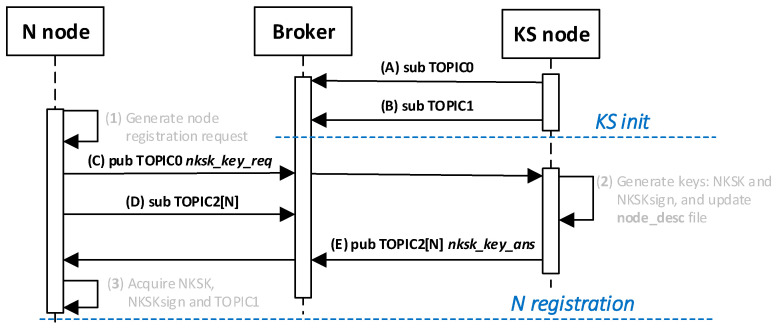
The sequence diagram of the data exchange in the Message Queuing Telemetry Transport (MQTT) service for the registration procedure.

The sequence diagram of the data exchange in the MQTT service for the registration procedure of the N node in the domain is shown in the Figure 17.

### 4.6. Procedure for Forwarding the List of Authorized Nodes to Cooperation

**Figure 18:**
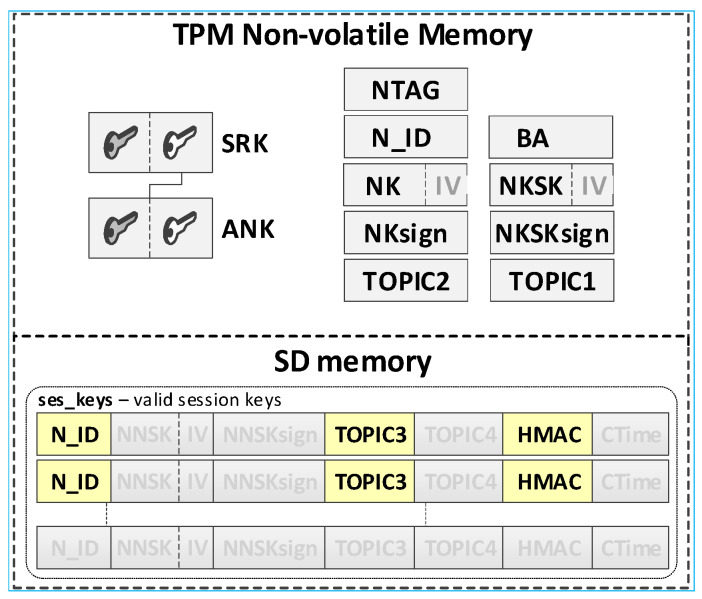
An example of data stored on the N node after the procedure in which two new nodes were added.

This procedure requires N node cooperation with an AC node. Similarly to the procedure for preparing the credentials for N nodes (described in Section 4.3), this procedure is not described here in detail, but only the expected results of this procedure are given. The result of this procedure is the appearance of new, incomplete entries in the file **ses_keys** stored in the resources of the N node. These new records contain only identifiers and TOPIC3 (the remaining fields are empty) for other N-type nodes with which the given node is authorized to establish a common symmetrical session key with those nodes. The list of such nodes is the basis for the procedure for generating session keys, a description of this procedure is given in the Section 4.7. An example of data stored on the N node after the procedure in which two new nodes were added is shown in Figure 18 (the updated data after adding two identifiers are highlighted in yellow).

### 4.7. The Procedure for Generating Session Keys

This procedure is intended to generate a new symmetric session key for a pair of N-type nodes (one element of the pair is a given node and the other element is another N-type node) and ensure secure transfer of the generated keys to another N node. The KS node is responsible for generating the session key and distributing it to the nodes concerned. The procedure consists of three stages:

(a) Requesting the session key;
(b) Providing the session key to the destination node;
(c) Confirmation of the delivery of the session key to the destination node.

The condition for the correct completion of the procedure is to register both nodes in the resources of the KS node before starting the procedure of generating the session key for these nodes. In the further description of the procedure, it was assumed that the key will be generated for the pair of N1 and N2 nodes. The designation N1 refers to a given node, which will initiate the session key generation procedure, and N2 refers to the second node from this pair of nodes. As a result of the procedure, the session key generated at the request of the N1 node and topics TOPIC3 and TOPIC4 will be securely transferred to the N1 and N2 nodes. Later, these topics will be used by the N1 and N2 nodes in the MQTT service during secure data exchange that will use the generated session key.

**Figure 19:**
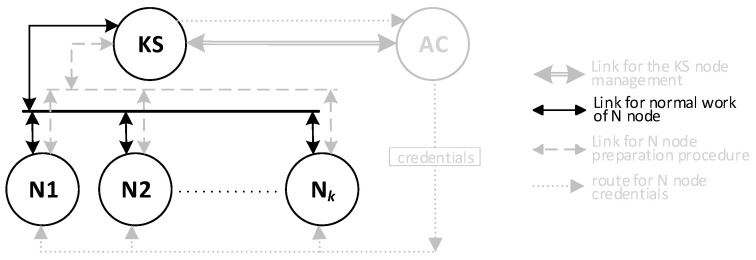
The way the KS node and the N node work together during the procedure for generating session keys.

**Figure 20:**
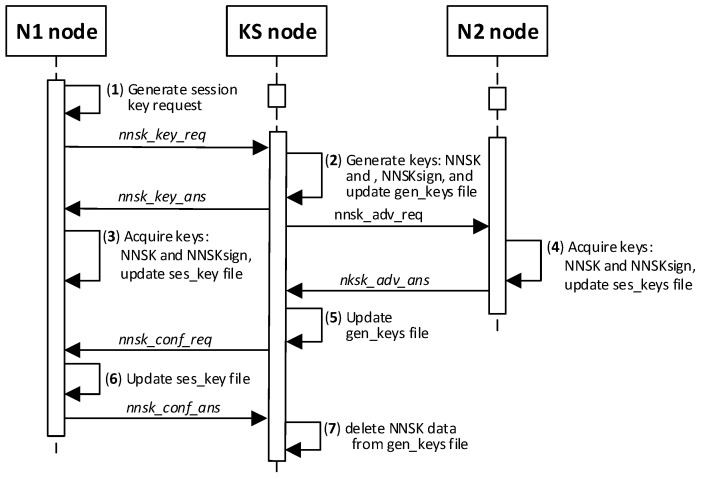
The sequence diagram for the procedure for generating session keys.

**Figure 21:**
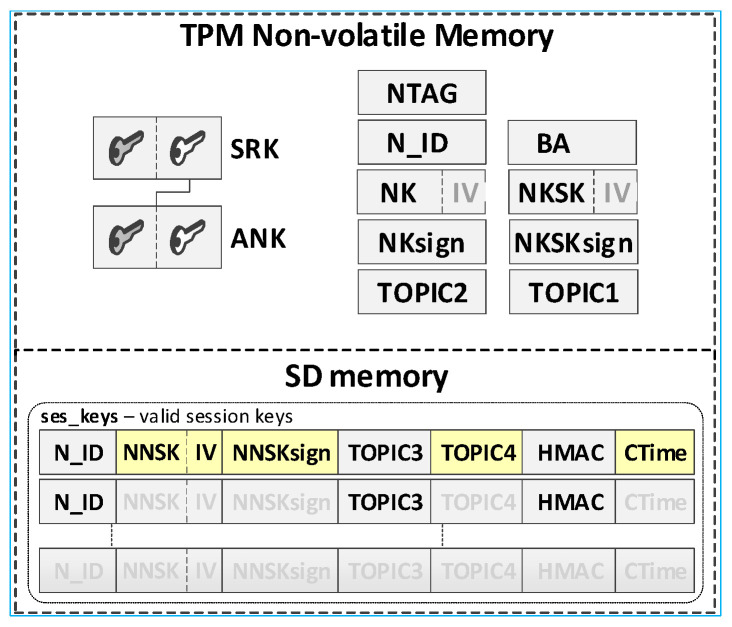
An example of the data stored on each N-type node after the procedure for generating session keys (the first entry in the ses_keys file is complete → the keys described there are known to N1 and N2).

**Figure 22:**
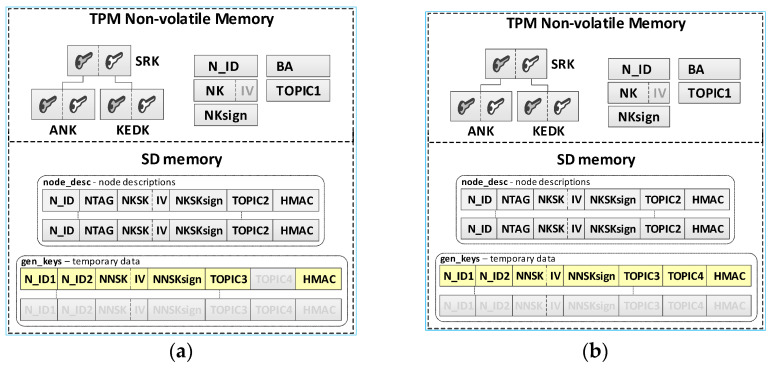
The content of data stored by the KS node after the step (3) (**a**) and after the step (5) (**b**).

The way the KS node and the N node work together during this procedure is shown in Figure 19. The sequence diagram for the procedure is shown in Figure 20, and an example of the data stored on each N-type node after the procedure is shown in Figure 21 (the updated data after generating sessions key are highlighted in yellow). The content of data stored by the KS node after step (2) and after step (5) is shown in Figure 22. After completing the procedure, in the gen_keys file stored by the KS node, there is no longer the entry regarding the generated key.

**Figure 23:**
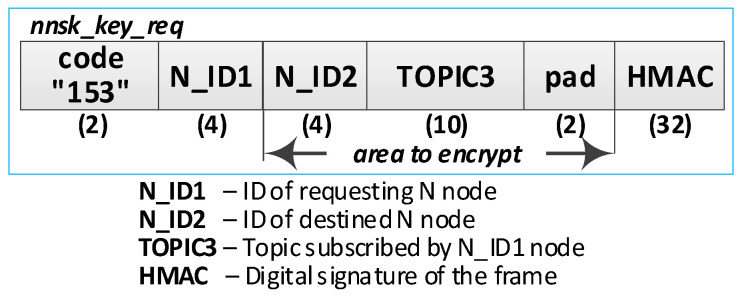
The session key request frame.

**Figure 24:**
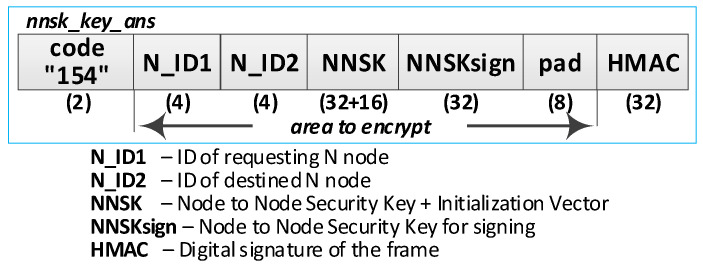
Response to the session key request frame.

**Figure 25:**
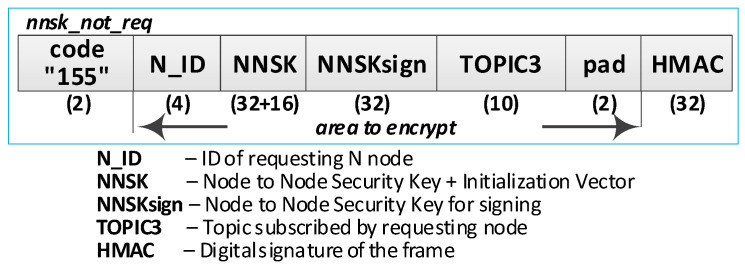
The frame of notification about the new session key.

**Figure 26:**
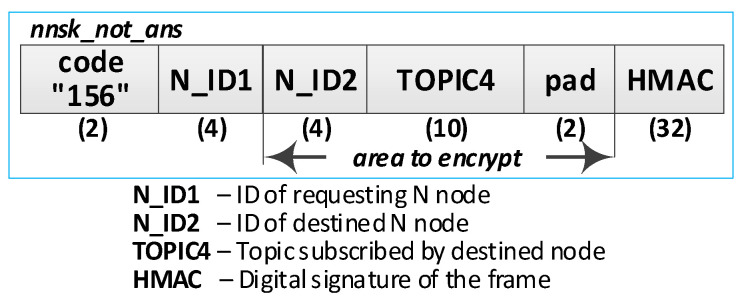
The response to frame of notification about the new session key.

**Figure 27:**
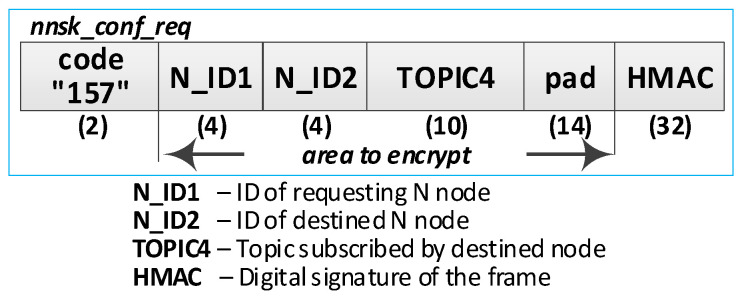
The frame of session key confirmation request.

**Figure 28:**
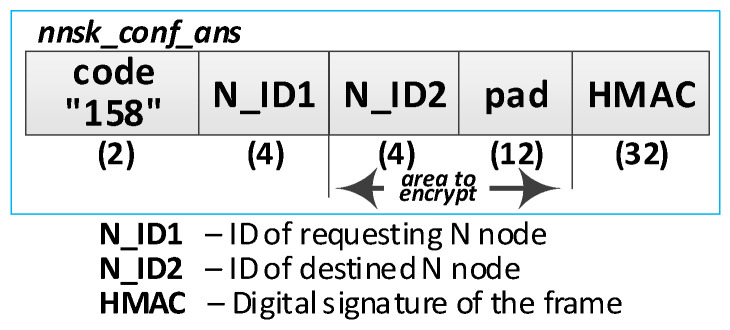
The response to the frame of session key confirmation request.

Detailed descriptions of the most important steps of the procedure (in Figure 20, the numbers in parentheses (e.g., (2)) preceding the descriptions indicate the numbers of the individual steps) are as follows:

**(1)**  **Generate session key request**. Conduct the following:
  - Prepare the ***nnsk_key_req*** packet (Figure 23) containing the following data: N_ID1, N_ID2, and topic TOPIC3 which will be subscribed by the N_ID1 node during secure data exchange with the N_ID2 node. The N_ID2 and TOPIC3 fields of the packet are encrypted using the NKSK of N_ID1 node, the HMAC is determined for all fields of the packet using the NKSKsign of the N_ID1 node;
  - Publish a ***nnsk_key req*** packet using the topic TOPIC1.
**(2)**  **Generate keys**: NNSK and NNSKsign and create a new entry in the **gen_keys** file. Conduct the following:
  - On the KS node, decrypt the data from the ***nnsk_key_req*** packet using the NKSK from the description of the N_ID1 node and verify the HMAC.
  - On the KS node, generate the keys NNSK with the initialization vector and NNSKsign, create the description of these keys, and then encrypt this description using the NK and the IV vector of the KS node; determine the HMAC using NKsign and append the keys description in local file **gen_keys**. The fields of the description should have the following values:
    N_ID1 = N_ID1 gathered from the ***nksk_key_req*** packet;
    N_ID2 = N_ID2 gathered from the ***nksk_key_req*** packet;
    NNSK and IV = value generated by the KS node;
    NNSKsign = value generated by the KS node;
    TOPIC3 = TOPIC3 gathered from the ***nksk_key_req*** packet;
    TOPIC4 = empty;
    HMAC = HMAC determined for all fields of the entry using NKsign key;
    CTime—time stamp of the operation;
    The fields NNSK, NNSKsign, TOPIC3, and TOPIC4 are encrypted using the NK key of the KS node.
  - Publish a response to the session key request frame to the N_ID1 node (***nnsk_key_ans*** packet showed in Figure 24) using topic TOPIC2 of N1 node. The fields: N_ID1, N_ID2, NNSK, and NNSKsign are encrypted using the NKSK of N_ID1 node; the HMAC is determined for all fields of the packet using the NKSKsign of N_ID1 node.
  - Publish a notification about the new session key to N_ID2 node (***nnsk_not_req*** packet showed in Figure 25) using topic TOPIC2 of the N2 node. The fields: N_ID, NNSK, NNSKsign, and TOPIC3 are encrypted using the NKSK of N_ID2 node; the HMAC is determined for all fields of the packet using the NKSKsign of N_ID2 node.
**(3)**  **On N1 node—acquire keys: NNSK and NNSKsign** and update the **ses_key** file. Conduct the following:
  - On the N1 node, decrypt the data from the ***nnsk_key_ans*** packet using the NKSK of the N1 node and verify the HMAC.
  - On the N1 node, update the entry for node N_ID2 in the **ses_keys** file based on the data from the received frame. The fields of the entry should have the following values:
    N_ID—should be the same as field N_ID2 from the received frame;
    NNSK and IV = NNSK gathered from the nnsk_key_ans packet;
    NNSKsign = NNSKsign gathered from the nnsk_key_ans packet;
    TOPIC3—should remain unchanged;
    TOPIC4 = empty;
    HMAC = HMAC determined for all fields of the entry using the NKsign key;
    The fields NNSK, NNSKsign, TOPIC3, and TOPIC4 are encrypted using NK key of the N1 node.
**(4)**  **On N2 node—acquire keys: NNSK and NNSKsign** and update **ses_key** file. Conduct the following:
  - On the N2 node, decrypt the data from the ***nnsk_not_req*** packet using the NKSK of the N2 node and verify the HMAC.
  - On the N2 node, create the entry for node N_ID1 in **ses_keys** file based on the data from the received frame. The fields of the entry should have the following values:
    N_ID–N_ID gathered from the nnsk_not_req packet;
    NNSK and IV = NNSK gathered from the nnsk_not_req packet;
    NNSKsign = NNSKsign gathered from the nnsk_not_req packet;
    TOPIC—10-bytes string generated by the the N2 node;
    TOPIC4 = TOPIC3 gathered from the nnsk_not_req packet;
    HMAC = HMAC determined for all fields of the entry using the NKsign key;
    The fields NNSK, NNSKsign, TOPIC3, and TOPIC4 are encrypted using the NK key of the N2 node.
  - Publish response to notification about the new session key to KS node (***nnsk_not_ans*** packet showed in Figure 26) using topic TOPIC1 of the KS node. The fields: N_ID2 and TOPIC4 are encrypted using the NKSK of the N_ID2 node; the HMAC is determined for all fields of the packet using the NKSKsign of N_ID2 node.
**(5)**  **Update gen_keys file**. Conduct the following:
  - On the KS node, decrypt the data from the ***nnsk_not_ans*** packet using the NKSK from the description of the N_ID2 node and verify the HMAC.
  - On the KS node, update the description of the session key requested by the N_ID1 node in the **gen_keys** file. The fields of the description should have the following values:
    N_ID1—should be the same as field N_ID1 from the received frame;
    N_ID2—should be the same as field N_ID2 from the received frame;
    NNSK and IV—should remain unchanged;
    NNSKsign—should remain unchanged;
    TOPIC3—should remain unchanged;
    TOPIC4 = TOPIC4 gathered from ***nnsk_not_ans*** packet;
    HMAC = HMAC determined for all fields of the entry using NKsign key of the KS node;
    The fields N_ID2, NNSK, NNSKsign, TOPIC3, and TOPIC4 are encrypted using the NK key of the KS node.
  - Publish a session key confirmation request to the N1 node (***nnsk_conf_req*** packet showed in Figure 27) using topic TOPIC2 of the N1 node. The fields: N_ID1, N_ID2, and TOPIC4 are encrypted using the NKSK of N_ID1 node; the HMAC is determined for all fields of the packet using the NKSKsign of N_ID1 node.
**(6)**  **Update ses_key file**. Conduct the following:
  - On the N1 node, decrypt the data from the ***nnsk_conf_req*** packet using the NKSK of the N1 node and verify the HMAC.
  - On the N1 node, update the entry for node N_ID2 in the **ses_keys** file based on the data from the received frame. The fields of the entry should have the following values:
    N_ID—should be the same as field N_ID2 from the received frame;
    NNSK and IV—should remain unchanged;
    NNSKsign should remain unchanged;
    TOPIC3—should remain unchanged;
    TOPIC4 = TOPIC4 gathered from the nnsk_conf_req packet;
    HMAC = HMAC determined for all fields of the entry using the NKsign key;
    The fields NNSK, NNSKsign, TOPIC3, and TOPIC4 are encrypted using NK key of the N1 node.
  - Publish a response to session key confirmation request to SK node (***nnsk_conf_ans*** packet showed in Figure 28) using topic TOPIC1 of the KS node. The field N_ID2 is encrypted using the NKSK of N_ID1 node, the HMAC is determined for all fields of the packet using the NKSKsign of the N_ID1 node.
**(7)**  **Delete NNSK data from gen_keys file**. Conduct the following:
  - On the KS node, decrypt the data from the ***nnsk_conf_ans*** packet using the NKSK from the description of the N_ID1 node and verify the HMAC;
  - Delete the session key entry for the N_ID1 and N_ID2 node pair from the **gen_keys** file.

**Figure 29:**
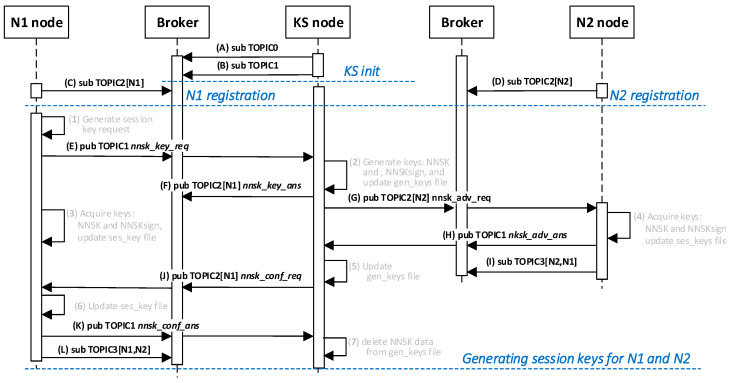
The sequence diagram of the data exchange in the MQTT service for the session key generation procedure.

The sequence diagram of the data exchange in the MQTT service for the procedure of generating the session keys for N1 and N2 nodes is shown in the Figure 29.

### 4.8. Procedure for Secure Data Exchange between Nodes

**Figure 30:**
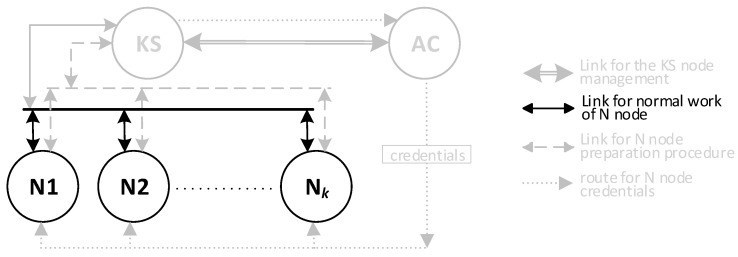
The way the N1 node and the N2 node work together during the data exchanging.

**Figure 31:**
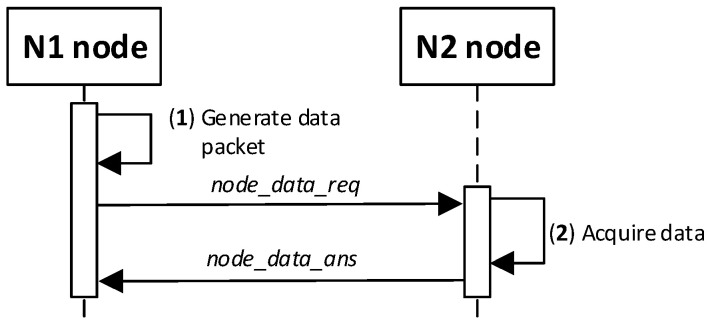
The sequence diagram for the procedure of exchanging data between nodes.

The data exchange protocol between the N1 and N2 nodes for which session keys were generated depends on how the two nodes cooperate and is established outside the key generation/renewal system. This topic is not the content of the study. For this reason, to demonstrate the operation of the KGR system, a simple transmission of a 12-byte character string from node N1 to node N2 will be shown as well as sending a response frame. The way the N1 node and the N2 node work together during this experiment is shown in Figure 30. The sequence diagram for the procedure is shown in Figure 31.

**Figure 32:**
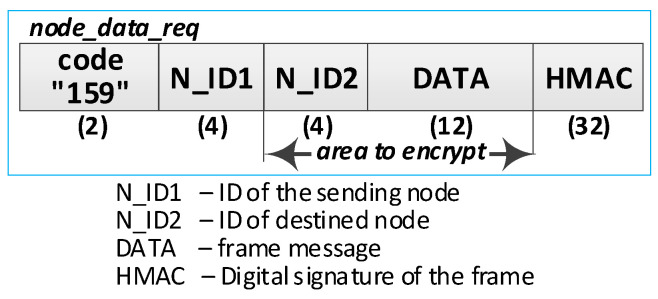
The data frame.

**Figure 33:**
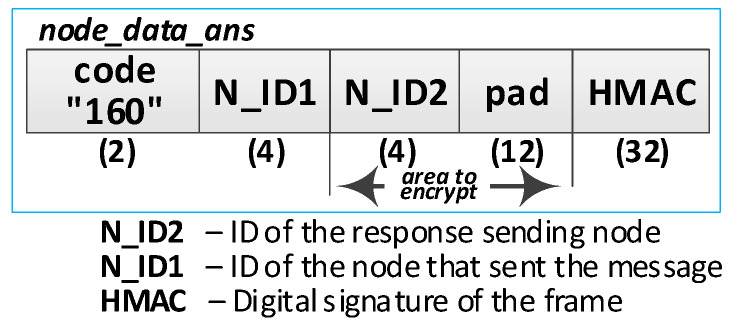
Response to the data frame.

Detailed descriptions of the most important steps of the procedure (in Figure 31, the numbers in parentheses (e.g., (2)) preceding the descriptions indicate the numbers of the individual steps) are as follows:

**(1)**  **Generate data packet**. Conduct the following:
  - Prepare the ***node_data_req*** packet (Figure 32) containing the following data: N_ID1 (ID of sending node), N_ID2 (ID od destination node), and DATA. The N_ID2 and DATA fields of the packet are encrypted using the NKSK session key common for N_ID1 and N_ID2 nodes; the HMAC is determined for all fields of the packet using also common NNSKsign;
  - Publish a ***node_data_req*** packet using the topic TOPIC3 subscribed by the N_ID2 node for exchange data session with N_ID1 node.
**(2)**  Acquire data. Conduct the following:
  - On the N2 node, decrypt the data from the ***node_data_req*** packet using the NNSK session key for a pair of nodes N1 and N2 and verify the HMAC.
  - Extract the data from the DATA field.
  - Publish a response to the data packet frame to the N_ID1 node (***node_data_ans*** packet showed in Figure 33) using topic TOPIC3 subscribed by the N_ID1 node for exchange data session with the N_ID2 node. The field N_ID2 is encrypted using the NNSK session key for a pair of nodes N1 and N2, the HMAC is determined for all fields of the packet using common NKSKsign.

**Figure 34:**
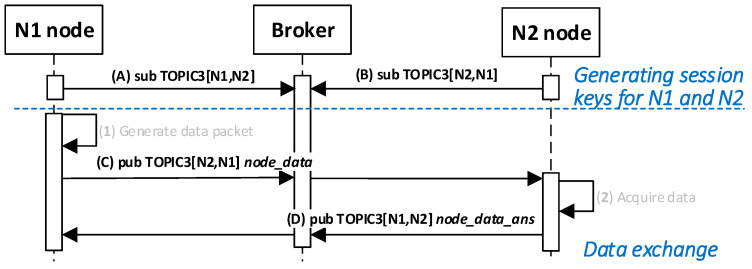
The sequence diagram of the data exchange in the MQTT service for the procedure of sending data from N1 node to N2 node.

The sequence diagram of the data exchange in the MQTT service for the procedure of sending data from N1 node to N2 node is shown in the Figure 34.

### 4.9. The Procedure for Renewing the Session Keys

Each cryptographic key loses its validity and must be renewed, regardless of how the cryptographic key was generated, what the key was used for, or how it was stored. Too long using the same key makes it easier to guess by unauthorized persons. A separate and very important problem is determining the criterion of validity of the cryptographic key. As a measure of the “*consumption*” of the key, you can use the passage of time from the moment the key is generated, the number of data portions (e.g., frames) secured with the key, or the size of the data (calculated in bytes) secured with the key, etc. The KGR system does not provide mechanisms for determining the key renewal criterion, and these issues are not the content of the study. In contrast, the KGR system is ready to renew keys upon request of N-type nodes.

This procedure is intended to renew invalid symmetric session key for a pair of N-type nodes and ensure secure transfer of the generated keys to both N nodes. The KS node is responsible for generating a new session key and distributing it to the nodes concerned. The procedure consists of three stages:

(a) Session key renewal request—includes the process of notifying the other party that the procedure has been initiated;
(b) Providing a renewed session key to the destination node;
(c) Confirmation of the delivery of the renewed session key to the destination node.

The KS node stores the generated keys during the key distribution process, and after sending the key to interested parties, all data related to this key are deleted from the resources of the KS node. From the KGR system point of view, the procedure for renewing the session key is very similar to the procedure for generating the session key. The only difference is that when generating a new key in the resources of N1 and N2 nodes, new entries are created in their ses_keys files containing the key attributes, and in the renewal procedure, the entries already exist but are filled with a new data. During key renewal, the following fields are modified: NNSK and NNSKsign keys as well as TOPIC3 and TOPIC4 fields.

Because the session key generation procedure is described in detail in the Section 4.7, and due to the similarity of both procedures, the procedure for renewing the session key will not be described in detail here.

## 5. Security Evaluation

Comprehensive security solutions for secure implementation of the Internet of Things should include preventive, detective, and reactive measures [28]. In the concept of the KGR system attention has been paid to preventive measures. The goal is to prevent and obstruct a certain group of typical attacks. This does not mean that the concept completely omits the use of detective and reactive measures.

Because the KGR system is mainly intended for IoT network nodes, which usually use wireless links to exchange data, the risks specific to such networks were considered in the security analysis. In most cases, sensor network nodes are unattended and can be an easy target for attack. For this reason, the following attacks will be considered: malicious sensor node injection (node replication attack), sensor impersonation (imitation), attacks on the information in transit, DoS attacks, and routing attacks [28]. The presented solution uses selected mechanisms offered by TPM to counteract these attacks. These include: securing cryptographic keys through a hierarchy of keys, creating a local trust structure, using PCR registers to detect attempts of unauthorized modification of data, software and hardware configuration of the sensor node, cryptographic protection of data stored in sensor node resources (in SD and NVRAM memory), and determining the value of the HMAC function for transmitted and stored data to ensure data integrity. The following sections also present what TPM mechanisms have been used for this purpose.

### 5.1. Node Replication Attack

The attack involves adding a cloned node to the network. For IoT nodes, this attack is relatively easy to perform when the node is unsecured. In the case of KGR system nodes, such an attack will not be possible, because the KS node and N-type nodes are equipped with TPM modules. Each TPM will use a symmetric SRK key that cannot be deleted or regenerated. This key is at the top of the key hierarchy, which creates a local trust structure. In this structure, each node has an ANK key, which is used to secure the cryptographic material stored in the node’s resources and to secure the cryptographic keys of the node. It has been assumed that the creation of a local trust structure on a node is conditioned by passing credentials to the new node. The prepared node can only obtain this data in a secure and controlled area via the AC node. It should be recognized that such an attempt will not be possible by unauthorized persons. Another way could be to try to obtain an ASK key from a working node. However, this attempt requires unauthorized interference with the node’s resources. These types of actions can be detected by the mechanism offered by the TPM module using its Platform Configuration Registers (PCR). If such actions are detected, then the node’s software may take various actions, e.g., turning off the device, deleting sensitive data, informing other nodes about such an incident, as well as physical destruction of the node. These types of activities are not the content of this study.

### 5.2. Sensor Impersonation

Impersonation of the sensor is very difficult to perform. Before starting normal operation, its node identifier (N_ID) and special tag (NTAG) are set for each node. These node credentials are stored in the node’s TPM resources and protected using TPM mechanisms. All these parameters are determined during the node preparation procedure and used during the domain registration procedure. The NTAG tag is of utmost importance. This tag is never passed in explicit form. Determining the value of this tag requires knowledge of the node identifier and other fields known only to the KS node.

### 5.3. Attack on Information in Transit

Data sent over wireless links are particularly susceptible to eavesdropping, modification, injection, interruption, and traffic analysis. The concept of the KGR system provides the use of authentication techniques and confidentiality and integrity checking of transmitted data. Each portion of data transferred will be supplemented with HMAC, which will use keys known only to individual pairs of nodes that exchange data. An additional safeguard, which is provided in the concept, is the generation of individual strings for topics used in the MQTT service. The content of each topic will also be known only for a pair of nodes that exchange data via the MQTT service. The distribution of these topics will take place during the secure distribution of cryptographic keys between nodes.

### 5.4. Denial of Service

DoS attack at the physical level and in the upper layers is beyond the scope of protection offered by mechanisms of the KGR system. To increase the system’s resistance to these types of attacks, you should use all known methods of securing your network against such attacks.

### 5.5. Routing Attacks

Many attacks choose the WSN routing protocol as their target. Almost all such attacks, such as Selective Forwarding, Sybil Attack, Wormhole Attack, Sinkhole Attack, or False Routing Information, require the placement of a malicious node in the network or manipulation of an existing node. The possibilities of performing such attacks in the KGR system are very limited. The mechanisms described in Section 5.1 and Section 5.2 counteract this.

### 5.6. Botnet Activities

Botnets are a significant threat to the IoT network. Botnets are designed to spread infection to misconfigured devices and then attack the target node after receiving the appropriate command from the person controlling the bot. An example of such a bot is Mirai malware [29]. Solutions proposed in the concept of the KGR system ensure resistance to the injection of bots by protecting its resources using the PCR registers of the TPM module. These issues are not considered in the study.

## 6. Implementation

**Figure 35:**
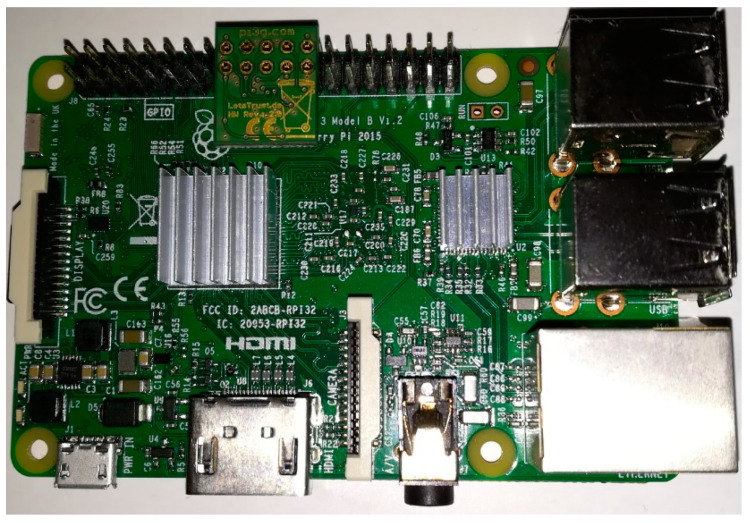
Raspberry Pi 3B+ with LetsTrust TPM v.2.0.

**Figure 36:**
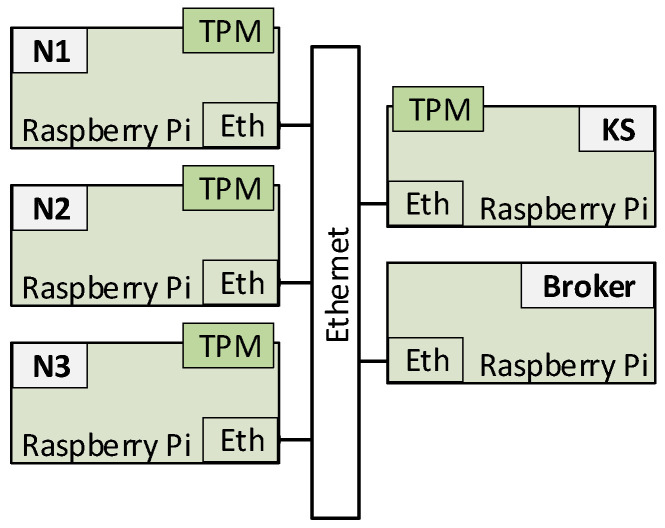
Structure of the simplified KGR system demonstrator.

The presented concept has not yet been fully verified. However, attempts were made to examine a very simple version of the cryptographic key generation system that met the selected requirements described at the beginning of Section 3.1. The design and demonstrator of this version were described in [30]. The prepared demonstrator included three N-type nodes (N1, N2, and N3), a KS node, and an MQTT broker node (AC node not implemented). All nodes were implemented using the Raspberry Pi 3 Model B board. Nodes N1, N2, and N3 and the KS node were additionally equipped with a LetsTrust TPM module containing the Infineon Optiga ™ SLB 9670 TPM 2.0 chip and 32GB SD memory (Figure 35). OASIS MQTT Version 3.1.1 was used to support the MQTT protocol. An Ethernet interface was used for data exchange. The structure of the demonstrator is shown in the Figure 36. The demonstrator software was prepared with the use of Python and the IBM TSS 1470 library.

The conclusions of the experiments were the basis for developing the presented concept and specifying the requirements for the system, which in addition to generating cryptographic keys should give the opportunity to renew the keys. Particular attention was paid to refining secure data exchange in all phases of the system life cycle.

## 7. Future Work

Future work will be focused on the implementation of the presented system and its testing. I predict that in the first stage of testing, the correctness of the solutions presented in the concept will be verified, and in the second stage, penetration tests for the system and testing of system resistance to various attacks will be performed. Further research stages will include the use of the KGR system for the secure exchange of data between secure domains of sensor nodes [7,8] and the extension of N-node preparation procedures for the case of hardware TPM v2.0, which implements all the features defined in the specification [25].

## Figures and Tables

**Table 1:** List of topics used by the nodes.

Topic	Node	Purpose
TOPIC0 ^1^	KS	for each N node for the first request during the registration procedure
TOPIC1	KS	for subsequent requests from the given N node during the registration procedure
TOPIC2	N	for requests from KS node
TOPIC3*_mn_*	N*_m_*	for requests from N*_n_* node
TOPIC4*_mn_*	N*_m_*	for publishing to N*_n_* node

^1^ Only TOPIC0 has fixed “*register*” content, the content of other topics is randomly generated.
